# ITFDS: Channel-Aware Integrated Time and Frequency-Based Downlink LTE Scheduling in MANET

**DOI:** 10.3390/s20123394

**Published:** 2020-06-16

**Authors:** Le Minh Tuan, Le Hoang Son, Hoang Viet Long, L. Rajaretnam Priya, K. Ruba Soundar, Y. Harold Robinson, Raghvendra Kumar

**Affiliations:** 1 Hanoi University of Home Affairs, Hanoi 01000, Vietnam; letuan104@gmail.com; 2 VNU Information Technology Institute, Vietnam National University, Hanoi 01000, Vietnam; sonlh@vnu.edu.vn; 3 Division of Computational Mathematics and Engineering, Institute for Computational Science, Ton Duc Thang University, Ho Chi Minh City 70000, Vietnam; 4 Faculty of Mathematics and Statistics, Ton Duc Thang University, Ho Chi Minh City 70000, Vietnam; 5 Department of Electronics and Communication Engineering, Francis Xavier Engineering College, Tirunelveli, Tamil Nadu 627003, India; priyawyn@gmail.com; 6 Department of Computer Science and Engineering, P.S.R.Engineering College Sivakasi, Tamil Nadu 626140, India; rubasoundar@gmail.com; 7 School of Information Technology and Engineering, Vellore Institute of Technology, Vellore, Tamil Nadu 632014, India; yhrobinphd@gmail.com; 8 Department of Computer Science and Engineering, GIET University, Gunupur 765022, India; raghvendraagrawal7@gmail.com

**Keywords:** mobile ad-hoc networks (MANETs), mobile computational grids (MCGs), frequency domain scheduling, long term evolution (LTE), largest weight delay first (LWDF)

## Abstract

One of the crucial problems in Industry 4.0 is how to strengthen the performance of mobile communication within mobile ad-hoc networks (MANETs) and mobile computational grids (MCGs). In communication, Industry 4.0 needs dynamic network connectivity with higher amounts of speed and bandwidth. In order to support multiple users for video calling or conferencing with high-speed transmission rates and low packet loss, 4G technology was introduced by the 3G Partnership Program (3GPP). 4G LTE is a type of 4G technology in which LTE stands for Long Term Evolution, followed to achieve 4G speeds. 4G LTE supports multiple users for downlink with higher-order modulation up to 64 quadrature amplitude modulation (QAM). With wide coverage, high reliability and large capacity, LTE networks are widely used in Industry 4.0. However, there are many kinds of equipment with different quality of service (QoS) requirements. In the existing LTE scheduling methods, the scheduler in frequency domain packet scheduling exploits the spatial, frequency, and multi-user diversity to achieve larger MIMO for the required QoS level. On the contrary, time-frequency LTE scheduling pays attention to temporal and utility fairness. It is desirable to have a new solution that combines both the time and frequency domains for real-time applications with fairness among users. In this paper, we propose a channel-aware Integrated Time and Frequency-based Downlink LTE Scheduling (ITFDS) algorithm, which is suitable for both real-time and non-real-time applications. Firstly, it calculates the channel capacity and quality using the channel quality indicator (CQI). Additionally, data broadcasting is maintained by using the dynamic class-based establishment (DCE). In the time domain, we calculate the queue length before transmitting the next packets. In the frequency domain, we use the largest weight delay first (LWDF) scheduling algorithm to allocate resources to all users. All the allocations would be taken placed in the same transmission time interval (TTI). The new method is compared against the largest weighted delay first (LWDF), proportional fair (PF), maximum throughput (MT), and exponential/proportional fair (EXP/PF) methods. Experimental results show that the performance improves by around 12% compared with those other algorithms.

## 1. Introduction

Industry 4.0 has now become reality with the support of advanced techniques in AI, distributed computing, and machine-to-machine (M2M) communication. One of the crucial problems in Industry 4.0 is how to strengthen the performance of mobile communication. In mobile ad-hoc networks (MANETs) and mobile computational grids (MCGs), devices communicate through the Wireless medium of mobile communication [1,2,3]. The 3G Partnership Program (3GPP) has introduced the fourth generation (4G)-long term evolution (LTE) technology that supports multiple users for downlink with higher order modulation up to 64 Quadrature Amplitude Modulation (QAM) [4]. It is a multi-user version of the popular orthogonal frequency-division multiplexing (OFDM) digital modulation scheme called orthogonal frequency-division multiple access (OFDMA). The OFDMA used in LTE has the advantages of MIMO-friendliness, scalable bandwidth and robustness against multi-path fading [5]. In LTE downlink transmissions, a high data rate up to 300 Mbps is achievable using a scalable bandwidth of 5 to 20 MHz [6,7,8].

The 3GPP specification defines that the downlink and uplink traffic are to be avoided by utilizing time division duplexing (TDD) and frequency division duplexing (FDD) [9]. However, multimedia applications strongly dominate LTE networks and may vary from live interaction and video conferencing to television. The resource scheduling in the LTE system for both uplink and downlink are managed by eNodeB and scheduled to various users deepening on the channel quality and service quality [10]. The domination of multimedia applications is due to their high transmission rate than any the other data formats. As the consequence, the traffic rate for mobile communications will be increased to a greater extent. Quality of service (QoS) should be guaranteed in terms of delay, quality and data loss by LTE scheduling for mobile multimedia applications [11].

QoS for any LTE system is directly dependent on the scheduling algorithm used. In the existing LTE scheduling, the scheduler in frequency domain packet scheduling exploits the spatial, frequency and multi-user diversity to achieve larger MIMO for the required QoS level [12]. For that, the scheduler considers channel conditions of all users upon possible MIMO modes to allocate the resource blocks (RBs) to users. There are several types of schedulers: channel unaware, channel aware or quality of service unaware and channel aware or quality of service aware [13]. The channel unaware schedulers are mostly used in wired networks, and their scheduling is performed by assuming the transmission medium as error-free and rigid. The channel aware/QoS unaware schedulers allocate the resources by analyzing the current channel conditions. Proportional fair (PF) [14] and PF-PF [15] are some of the channel aware/QoS unaware schedulers. PF-PF is the joint time and frequency scheduler. The channel aware/QoS aware scheduler considers both the channel quality and QoS required by user applications. It is mostly used in LTE systems to meet their performance requirements. The M-LWDF and EXP/PF are some channel aware/QoS aware schedulers [16].

Regarding time frequency scheduling, two levels of LTE scheduling for temporal and utility fairness were given in [17]. Later, earliest deadline scheduling was proposed in [18] for managing queues in the time domain. Packet prediction was emphasized in [19] to manage users in the time domain. In packet prediction, virtual queues are used to manage the queues under traffic. A scheduling algorithm for real-time traffic flow, which mainly concentrates on queue management and bandwidth allocation, was recommended in [20]. In LTE, a MAC scheduler sitting just above the physical layer [21] was used to categorize data packets that allocate resources upon traffic. The disadvantage of this method is the MAC scheduler allocates maximum resources to the GBR queue so non-GBR packets may suffer time delays.

OFDMA [22] was used for allocating resources in which the downlink channel in the period domain is separated into frames of 10 ms and every channel contains 10 subframes of 1 ms. The scheduling algorithms were classified as QoS-aware scheduling and QoS-unaware scheduling [23]. The maximum throughput scheduling leads the aggregation throughput according to the CQI to achieve high data RTE in the channel [24]. In fair scheduling algorithms, resource allocation would take placed by considering both the available data rate and the achieved data rate. It shows the trade-off between throughput and fairness [25]. A mechanism [26] accepting the concept [27] introduces a utility function for improving the fairness index. Scheduling based on optimal DRX embedded in the same MAC scheduler was given in [16]. Resource allocation for QoS-aware channels was emphasized in [28]. Throughput and delay can be calculated together in real-time and non-real-time allocations [29]. Depending upon the delay and channel quality, packets are scheduled to the physical layer for transmission [30]. 

**Figure 1:**
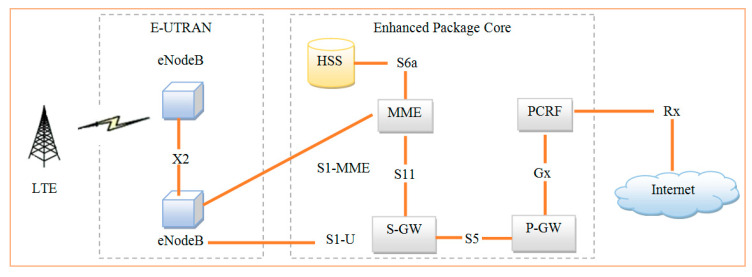
Architecture of LTE - 3GPP.

LTE networks provide very high data rates for downlink transmissions so that they can transmit large amounts of data with packet optimization to users [31]. The base station is responsible for allocating resources to users’ equipment for LTE downlink transmission [32]. The downlink scheduling with delay-related weighted technique using LTE cellular networks provides better tradeoff of fairness and throughput between users in dissimilar traffic situations [33]. Dynamic packet scheduling is the main element of LTE network aiming to increase throughput and QoS to end users. Because of resource limitations, the queuing-based monitoring technique was used in downlink LTE communication to increase throughput and fairness among users [34]. The energy-based spectrum resources are optimized using the green cellular system to acquire the channel data for transmitting packets through base station [35]. The optimized resources are utilized to provide QoS at the downlink scheduling. A packet prediction technique was implemented in the eNodeB for optimization [36]. A channel-related feedback method [36] using the adaptive feedback threshold was generated to minimize the overhead while transmitting packets [37]. The system performance was measured using the downlink packet scheduling procedure [38]. The architecture of LTE network by 3GPP is shown in Figure 1.

3GPP is the universal mobile communication standards organization that works to provide the latest technology in order to reduce the traffic and identified the LTE standard release 8. LTE contains two subnetworks called the evolved-universal terrestrial radio access network (E-UTRAN) and evolved package core (EPC). E-UTRAN was introduced within LTE and it act as the interface between user equipment (UE) and eNodeB. LTE communicates by orthogonal frequency division multiplex (OFDM) with huge amount of orthogonal subcarrier signals to carry data on several parallel data stream and channels. The time domain has a 10ms radio resource in a frame and 10 subframes of 1ms length for each. In the other hand, the frequency domain has multiple subcarriers with 15KHz bandwidth for each. Around 12 subcarriers from the frequency domain and 0.5ms from time domain is regarded as a resource block (RB), which is allocated to users every 1ms called transition time interval (TTI). 

Figure 1 illustrates the LTE, which is divided into two parts, namely EPC and E-UTRAN. E-UTRAN has a flat architecture consisting of two eNodeB nodes, which will do all processing through an air interface protocol. It is an independent entity. One eNodeB is connected with the multiple eNodeB through interface X. The eNodeB is connected to the serving gateway (S-GW) through interface S1-U. Similarly, the serving gateway is connected to MME through interface S11. MME is responsible for control plane signaling in which the serving gate is responsible for the control plane or actual dataflow. MME is connected to a database called home subscriber server (HSS), which is a central database containing all the information like data rates required by the user, security keys of the users, etc. PDN gateway is the interface of the mobile to the external world as PCRF. 

Scheduling and resource allocation of uplinks and downlinks is performed in eNodeB. The resource allocation procedure is called scheduling process executed on MAC layer. 3GPP has been involved to produce the scheduling process in LTE applications that allow the service provider to discover the appropriate scheduling procedure. The system management has been measured using the effective scheduling algorithm. Since 3GPP released the updated standards beyond 15 to utilize with additional functionality, the new releases are more suitable for Industry 4.0 rather than the early development 5G versions [39].

The proposed LTE networks are constructed for improved coverage and infrastructure. They also support automatic connection through machine-to-machine (M2M) communication. LTE has improved mobile broadband communication, virtual reality and cloud-oriented services with peak data rates. It provides solutions for the connectivity problems occurring in several applications like smart cities, automated agriculture, transport system automation, the industry-based sensing grids, Internet of Things-related mines, energy-based industries, the construction field, machinery, airport automation, railway networks, robot-assisted hospitals and industry with closed loops. The shared spectrum models are used to allow building the integrated networks that are connected with Industry 4.0 through LTE models. ITU has established the reliable connectivity to maintain the communication. 5G technology is the next generation of 4G proposed as a standard for cellular networks in which the service area is divided into small geographical areas called cells. 5G user equipment connects to the telephone network and internet by radio waves through antennas in cells. The new network provides high bandwidth, allowing faster download speeds, eventually up to 10 Gbps, and offers a big advantage in Industry 4.0 but it is still in the research and development phase. Nowadays, just a small number of countries have deployed 5G successfully, while the rest are still using 4G or its predecessor, so that 4G LTE still has a big chance in Industry 4.0. The proposed method will be infrastructure for developing new methods applied for 5G networks in the future.

In this paper, we propose a channel-aware integrated time and frequency-based downlinks LTE scheduling (ITFDS) algorithm. The proposed scheduling algorithm distributes resources to all users together in the time and frequency domains to reduce convolution. It works well for real-time flows and also maintains fairness among users. The new algorithm is experimentally validated against related ones. The main contributions of this paper are as follows:

✓ Scheduling can be implemented using the propositional pair and throughput. It is used to compute the packet loss ratio and packet delay;
✓ The dynamic class-based establishment algorithm is used to solve the optimization problem for queue-based transmission;
✓ Scheduling is used to increase the network throughput by guaranteed rate for all devices;
✓ Time frequency LTE scheduling provides the temporal and utility fairness to achieve the required quality of service level;
✓ The proposed channel-aware integrated time and frequency-based downlink LTE scheduling algorithm is capable of both real-time and non-real-time applications;
✓ The optimization is achieved by providing the highest priority component with an urgent queue and scheduling process;
✓ Every user equipment packet is used for performing the scheduling process into the queue for transmission with the proposed LTE downlink scheduling technique;
✓ The performance analysis of the proposed algorithm is demonstrated in terms of packet delay, packet loss ratio, aggregated throughput and fairness index.

The remainder of this paper is organized as follows: Mathematical models followed by the results and discussion are presented in Section 2 and Section 3, respectively. Finally, conclusions are provided in Section 4.

## 2. Proposed Method

### 2.1. Main Ideas

The proposed LTE networks utilizing the downlink scheduling are constructed for improved coverage and infrastructure. It also supports the automatic connection through machine-to-machine communication. Shared spectrum models are used to allow for building the integrated networks that are connected with Industry 4.0 through LTE models. ITU has established the reliable connectivity needed to maintain the communication. 

The proposed algorithm is constructed in both the time and frequency domain in order to increase the quality of service (QoS) for mobile network users. The edge users experience enhanced throughput and reduced amounts of delay. The main issue for edge users is the worse channel condition because of the obstacles within the UEs and eNodeB. The scheduler is capable of analyzing the data about the delay and size of the packet of the users to facilitate the allocation of the resource blocks. Whenever a user is in communication with several eNodeB, its data could be transmitted using the superior quality channel for establishing capability aiming to increase channel quality. The scheduler separates UEs in spite of the level of the traffic. There are two groups: primary group and additional group. If the traffic is very high then the primary group is activated; otherwise the additional group is activated. According to the type of incoming packet, the quality of service maintains the priority-related scheduling technique. The optimization has been performed using the efficient scheduling algorithm where the highest priority component has the urgent queue for providing optimization. OFDM has been involved for the downlink communication procedure for providing 3GPP LTE. The multi-carrier transmission and frequency division multiplexing (FDM) are the main parameters for better bandwidth allocation. The scheduler is indeed an important component for assigning and sharing the physical resources between different kinds of users.

### 2.2. Model Formulation

**Figure 2:**
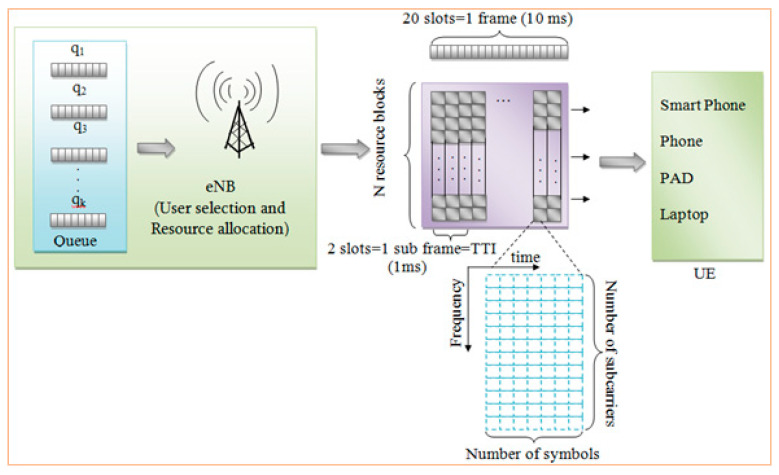
The simplified LTE network model.

Table 1 shows the notions and symbols used throughout the paper. A simplified model of LTE is shown in Figure 2, in which eNodeB consists of user selection and resource allocation. There are k numbers of queues consisting of q_1_, q_2_,…,…, q_k_ for the scheduling process. The queue list sends information to the eNodeB network. Then, the signal sends 20 slots of data with every frame consisting of several subframes with 1 ms time-to-live time span. The signal can be sent to external devices like smartphones, mobile phones, PADs and laptops. The scheduling can be done using two methods, namely frequency-based scheduling and time-based scheduling. The frequency is received into every subframe with n numbers of subcarriers. Each carrier can receive the signal and perform the scheduling process.

For real time traffic, maximum weight largest delay first (MWLDF) and exponential-proportional fair (EXP/PF) are introduced. In multi-user scheduling, distribution of resources among users is done by sharing the channel capacity among them. The channel capacity is calculated by using Shannon’s capacity theorem. LTE downlink transmission is based on OFDM modulation.

In the frequency domain, subcarriers are spaced at 15KHz each and with different modulation codes and rates. The modulation and coding are choosing according to the channel condition. A resource block (RB) consists of 12 subcarriers. In the time domain, a resource blocks contain one TTI which includes two time slots. Each time slot has six or seven OFDM symbols. Since *Cy(n)_i,k_* is the transmission rate, ${\left(n\right)}_{i,k}$ is nth TTI of the metric of *i*th user on *k*th RB, we have:

(1) $$Cy{\left(n\right)}_{i,k\;}=log_{2\;}\left[1+SINR{\left(n\right)}_{i,k}\right]$$

where, *Cy(n)_i,k_* is the maximum limit of the transmission rate. It is normally not equal to the actual *Cy(n)_i,k_*. In order to get maximum throughput and to allocate each RB to users with maximum channel capacity, the maximum throughput (MT) algorithm is used. The metric is expressed as:

(2) $$R_{i,k}^{MT}\left(n\right)=Cy{\left(n\right)}_{i,k}$$

Here, $R_{i,k}^{MT}\left(n\right)$ is real-data of *k*th user in *i*th time slot using the MT algorithm, Equation (2) satisfies non-real-time applications. If the flow is real-time data, we have:

(3) $$R_{i,k}^{MT}\left(n\right)=argmax_{i}C_{i,k}\left(t\right)$$

where $C_{i,k}\left(t\right)$ represents the current channel condition. The MT scheduling guarantees that the resource blocks are used to transmit the maximum data for maximizing the throughput in a good channel condition. However, users with poor channel condition are not allowed to use the channel.

The proportional fair scheduler concentrates on both available data rate and achieved data rate while calculating throughput:

(4) $$R_{i,k}^{PF}\left(n\right)=argmax_{i}\frac{C_{i,k}}{{\bar{C}}_{i}}\left(t\right)$$

Here, ${\bar{C}}_{i}$ represents the mean data rate for the *i*th user, which can be accurately calculated only for non-real time scenarios. Argmax is used to find the best channel condition for transmission. The transmission rate is computed through the packet waiting delay with the queue length for computing from the user equipment, so the transmission rate is equal to the user. The scheduler provides balance between fairness and the overall system throughput. Different scheduling mechanism correspond to different computations of the metric $C_{i,k}\left(t\right)$. For the concerned channel aware-QoS unaware case $C_{i,k}\left(t\right)$ is set based on the CQI feedback that gives the Signal-to-Interference-plus-noise ratio (SINR) of each user on each RB. The MT scheduling guarantees that each resource block can be used to transmit the maximum amount of data so that it is able to maximize the aggregate throughput. However, for users with poor channel conditions it is difficult to obtain available resources, and MT is unfair. For real time applications, the delay is calculated and multiplied with the utility factor to increase the fairness index:

(5) $$R_{i,k}^{\frac{EXP}{PF}}\left(n\right)=argmax_{i}\propto_{i}\frac{C_{i,k}}{\bar{C_{i}}}{\left(t\right)}_{\;\;}W_{i\;}\left(t\right)$$

Equation (5) describes the improvement version of the proportional fair scheduling algorithm. At the same TTI, the queue management is taken placed in the time domain. To improve the throughput, the head of delay is included for all users as:

(6) $$R_{i,k}^{MLWDF}\left(n\right)=argmax_{i}\;(\propto_{i}\frac{C_{i,k}}{\bar{C_{i}}}{\left(t\right)}_{\;\;}\exp\;(\propto_{i}\;\frac{W_{i\;}\left(t\right)-\bar{W_{i\;}\left(t\right)}}{1+\sqrt{W_{i\;}\left(t\right)}}))$$

If only one user is allowed to transmit at one time slot, this will degrade performance of the system. Proportional fair scheduling aims to make the tradeoff between multiuser diversity gain and fairness. A scheduling policy $R_{i,k}^{\frac{EXP}{PF}}$ is proportionally fair, if and only if the sum of the logarithmic average user throughput is maximized after the scheduling decision where *i* is the active user and $C_{i,k}$ is the active data rate. Based on the proposed formulae and notions, we introduce the new ITFDS in the next section.

### 2.3. Proposed ITFDS Scheduling Algorithm

The QoS requirement of real-time applications is based on the scheduling of resource blocks (RBs) based on priorities, channel capacity and the previous servicing rates of all users. The eNodeB collects CQI and QCI information from each piece of user equipment (UE). Depending upon the collected information, eNodeB allocates the resources to various UEs. The value of ∆ is computed from the total amount of bits in the data packets into the transmission buffer of the eNodeB with the flow of the data transmission in head of line.

**Algorithm 1** ITFDS Algorithm*Step 1*: Calculate the maximum capacity using Equation (1).*Step 2*: Generate the Channel Quality Indicator (CQI) in terms of data rate for different applications (Table 2).*Step 3*: According to QoS Class Identifier (QCI), we choose data rate for transmission (Table 1).*Step 4*: For high data rate transmission, we calculate ∝_i_ with Head of Line (HOL) Delay ∆_i,k_*Step 5*: Apply the Integrated Time and Frequency Domain Scheduling using Equation (7):
(7)$$R_{i,k}^{ITFDS}\left(n\right)=argmax_{i}\;(\propto_{i}\frac{C_{i,k}}{\bar{C_{i}}}{\left(t\right)}_{\;\;}\exp\;(\propto_{i}\;\frac{W_{i\;}\left(t\right)+\Delta_{i,k}\left(t\right)-\bar{W_{i\;}\left(t\right)}}{1+\sqrt{W_{i\;}\left(t\right)+\Delta_{i,k}\left(t\right)}}))$$
*Step 6*: In same TTI, queue manage is done in time domain by checking W_i_(t)+Δ_i,k_(t)≫W_i_(t). *Step 7*: Compute the value of $Q_{i,k}$ using Equation (8)
(8)$$Q_{i,k\;}=Q_{l_{i,k}\;}-Q_{th_{i,k}\;}$$
*Step 8*: if Q_i,k_ ≠ ∅, repeat steps 4 to 8*Step 9*: Calculate Packet Loss Rate (PLR), Packet Delay, Throughput and Fairness Index

The proposed scheduling algorithm is a channel-aware/QoS-aware scheduling algorithm (Algorithm 1). The channel conditions and QoS are included in scheduling parameters to distribute resources to all available users. In all TTI, eNodeB gathers information from various queues connected with it using $Q_{i,k}=Q_{l_{i,k}}-Q_{{\mathrm{th}}_{i,k}}$. User equipment (UE) calculates CQI using the reference signal from eNodeB and sends it back to eNodeB. The CQI is the quantized form of signal-to-interference-noise-ratio (SINR) [16]. A default classifier is found when a UE joins the system until the connection terminates. The classifiers are divided into guaranteed bit rate (GBR) and non-guaranteed bit rate (non-GBR) based on the quality of service constraint [17]. The QoS class identifier (QCI) helps to identify the type based on the application data of the UE.

**Figure 3:**
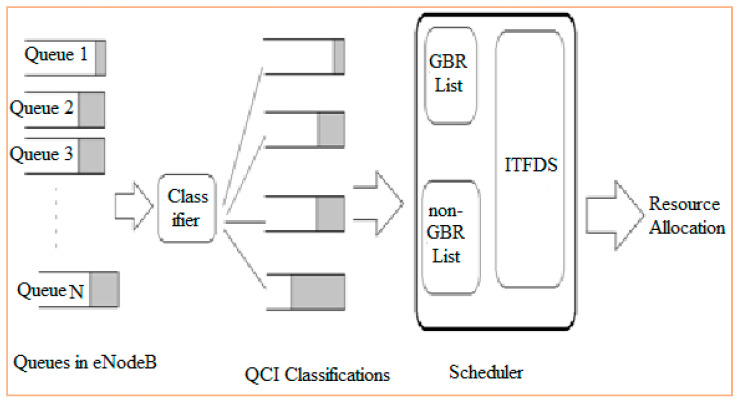
Block diagram of the proposed ITFDS.

**Figure 4:**
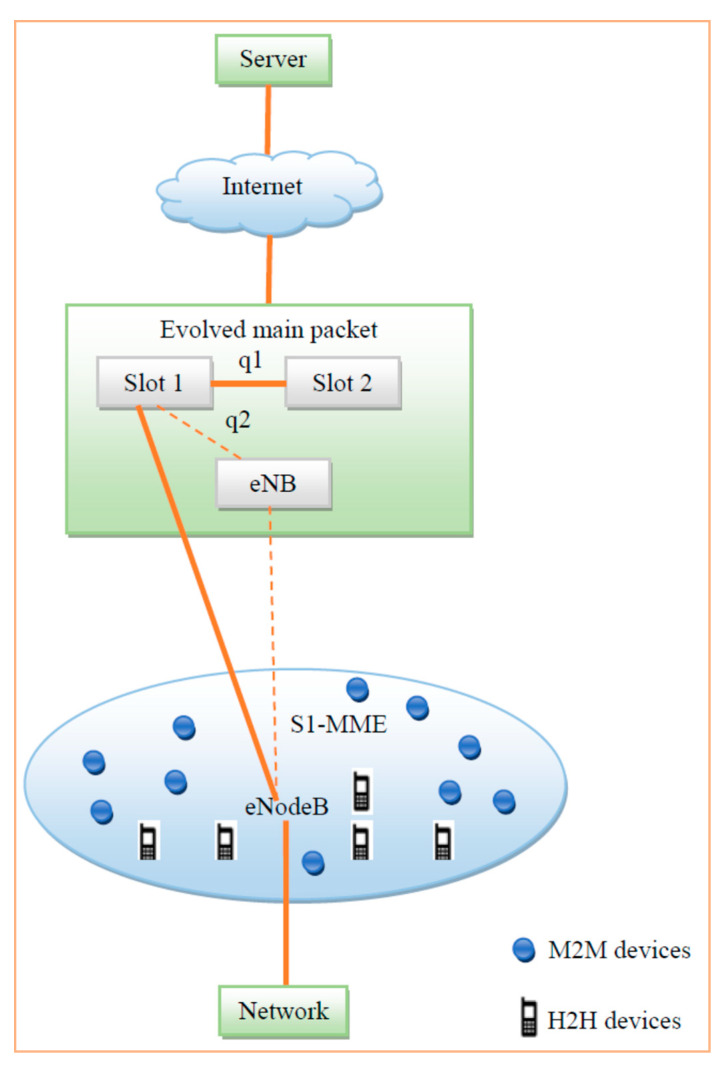
LTE architecture.

Figure 3 shows a block diagram of ITFDS. At eNodeB, a buffer is assigned to each UE. Packages arriving at the buffer are time stamped and queued for transmission as first-in-first-out (FIFO). Once the packets arrive, the classification of data can be done by a classifier (e.g., support vector machine – SVM [40]), which segregates arrived packets into real time and non-real time data with the help of CQI, then pushes the packets into the appropriate RT or NRT queues. After that, the proposed scheduling algorithm will schedule the packets in both the RT queue and the NRT queue as suitable for simultaneous parallel transmission according to the requirements of the algorithm. The proposed ITFDS scheduling is a channel-aware/QoS-aware scheduling algorithm. The channel conditions and QoS are included in scheduling parameters to distribute resources to all available users. In all TTIs, eNodeB gathers information from the various queues connected with it. The classifier is used to classify the traffic of different queues when a UE joins the system until the connection terminates. The QoS class identifier (QCI) helps to identify the type based on application data of UE. S1-MME is a single interface within the eNodeB and MME. The traffic parameters are divided into the H2H devices and M2M devices. H2H devices have the applications like voice browsing, IMS signaling and Multimedia applications. M2M devices have the applications like surveillance cameras, regular monitoring and emergency applications. The S-GW provides the data delivery of non-guaranteed applications and P-GW provides the internet-based operator services. Figure 4 demonstrates the LTE architectural model which consists of human-to-human (H2H) and machine-to-machine (M2M) devices for communication purposes. The eNodeB and evolved main packet are connected to the server via the internet using S1-MME.

### 2.4. Dynamic Class-based Establishment (DCE)

The symbols used for this proposed systems that $T,{\;T}_{1},{\;T}_{2}$ are the matrices, r, s are the elements of the transmission time interval, δ is the transmission state probability, k denotes the state, γ is the constant value, R denotes the resultant length, Q is the queue value, ${\mathrm{delay}}_{i}$ denotes the delay value, $P_{c}$ denotes the parameter of channels, $w_{k}$ is the weighted value, $T_{w}$ denotes the transmission range for the weighted value, $W_{i}\left(t\right)$ denotes the weighted time domain value in TTI, $Q_{l_{i,k}}$ denotes the queue length between the user *i* and *k*, $Q_{{\mathrm{th}}_{i,k}}$ denotes the threshold value of the queue size of the users *i*, *k*, $T_{v}$ denotes the transmission rate for user v, $T_{\mathrm{beginning}}$ is the transmission range in the beginning of the dynamic class-based establishment process.

Transmission Time Interval (TTI) can be created by using the matrix T_1_ and T_2_:

$$T=T_{1}\;\cup \;T_{2}$$

(9) $$T_{1}=\left(\begin{array}{c} \begin{array}{cc} 1-s\;\;\;\;\;\;\;\;\;\;\;\;\;\; & s\\ r\left(1-s\right)\;\;\;\;\;\;\;\;\;\;\;\; & rs+\left(1-r\right)\left(1-s\right) \end{array}\;\;\;\;\;\;\begin{array}{cc} 0 & 0\\ s\left(1-r\right) & 0 \end{array}\;\;\;\;\;\begin{array}{cc} 0 & \ldots .\\ 0 & \ldots . \end{array} \end{array}\right)$$

(10) $$T_{2}=\left(\begin{array}{c} \begin{array}{cc} 0\;\;\;\;\;\;\;\;\;\;\;\;\; & r\left(1-s\right)\\ \ldots .\;\;\;\;\;\;\;\;\;\;\;\; & \ldots . \end{array}\;\;\;\;\;\;\begin{array}{cc} rs+\left(1-r\right)\left(1-s\right) & r\left(1-s\right)\\ \ldots . & \ldots . \end{array}\;\;\;\;\;\begin{array}{cc} 0 & \ldots .\\ \ldots . & \ldots . \end{array} \end{array}\right)$$

The transmission condition is:

(11) δ T=δ

where:

(12) $$\delta =\left[\delta_{0},\delta_{1},\delta_{2},\;\ldots \ldots \ldots ,\;\delta_{k}\;,\ldots \ldots \right]$$

where $\delta_{k}$ is the transmission state probability of the state *k*. Equation (11) can be modified as

(13) $$\left[\delta_{0},\delta_{1},\delta_{2},\;\ldots \ldots \ldots ,\;\delta_{k}\;,\ldots \ldots \right]\;\left[T\right]=\left[\delta_{0},\delta_{1},\delta_{2},\;\ldots \ldots \ldots ,\;\delta_{k}\;,\ldots \ldots \right]$$

By solving Equation (12), we get:

(14) $$\delta_{1}=\gamma \;\delta_{0}\;,\delta_{2}=\gamma^{2}\;\delta_{0},\;\ldots \ldots \ldots ,\;\delta_{k}=\gamma^{k}\;\delta_{0}$$

where:

(15) $$\gamma =\frac{r\;\left(1-s\right)}{s\;\left(1-r\right)}$$

Finally:

(16) $$\delta_{0}+\delta_{1}+\delta_{2}+\ldots \ldots \ldots +\delta_{k}+\ldots +\delta_{\infty }=1$$

After simplifying Equation (15), we get:

(17) $$\delta_{0}=1-\gamma$$

(18) $$\delta_{0}=\frac{\left(s-r\right)}{s\;\left(1-r\right)}$$

The resultant length of the class in the queue is

(19) $$R\left(Q\right)=\overset{\infty }{\underset{k=1}{\sum }}k\delta_{k}$$

The length of the queue is related to resultant length of the class. It satisfies the following condition:

(20) $$R\left(Q\right)\;\leq delay_{i}$$

Additionally, the constraints for the optimization problem are:

(21) $$\{\begin{array}{l} R\left(Q\right)\;\leq delay_{i}\\ s_{i}\geq \;s_{i}+1\\ s_{i}\geq r_{i}\\ s_{1}+s_{2}+\ldots +s_{n}\leq \;1 \end{array}$$

The DCE algorithm is used to allocate the queue-based transmission from the group of non-identified TTI (Algorithm 2), the optimization is achieved using the dynamic class-based establishment algorithm that the data broadcasting has been maintained with the transmission range.

**Algorithm 2** Dynamic Class-based Establishment (DCE) algorithmInput: Group of non-identified TTIOutput: Allocation of queue-based transmission*Step 1*: Initialize the parameter of channels *P_c_,*$T_{beginning}$ is the Transmission range while transmission begins in the network*Step 2*: Calculate the highest value of transmission rate *T_v_**Step 3*: Determine the size of the TTI
(22)$$T_{w}=\overset{n}{\underset{k=1}{\sum }}w_{k}*{\left(P_{c}-T_{v}\right)}^{2}$$
*Step 4*: Calculate the weight of beginning transmission range for every iteration according to $P_{c}\&T_{w}$
(23)$$T_{current}=\overset{n}{\underset{k=1}{\sum }}w_{k}*{\left(P_{c}-T_{w}\right)}^{2}$$
*Step 5*: Compute the transmission range by$if\;(T_{beginning}>T_{current})$ then fix transmission range as $T_{beginning}$*Step 6*: Change the transmission range as
$T_{beginning}=T_{current}$


The DCE scheduling mechanism is executed as the beginning of the scheduling event. Here by metric weights of all $w_{k}$ on the RB i ∈ K are determined. Although there are different formulas to handle various flows QoS, although various flows QoS δ is calculated only once for all the available flows at the current TTI. After that, flows are compared with each other to select the flow j∈K with the highest metric. The possible overhead, in this case, is as a result, the overhead complexity to execute DCE in a TTI.

### 2.5. QoS Class Identifier (QCI) and Channel Quality Indicator (CQI)

In time-based and frequency-based scheduling, the scheduler first selects the user based on its QoS requirement along with fairness and decides its corresponding RBs proportion according to past average throughput achieved and queue length to meet the QoS requirement. For considering QoS requirement and fairness, the LWDF and the past occurred packet loss rate is used. For each user selected in time domain, the frequency domain scheduler allocates the required RBs portion in the channel bandwidth according to current channel conditions. The QCI value for various applications is given in Table 2 The standard CQI for various applications are given in Table 3. The following tables list the QoS class identifiers (QCIs) and channel quality indicators (CQIs) used in the proposed algorithm.

The fairness index measures that the packet is transmitted within the specified time. The user fairness indeed has been increased. Otherwise, it is incremented according to the communicated data separated by the size of the packet. Transmission time interval (TTI) is measured by separating the packet size of a particular user to achieve the throughput. If the user’s packet is smaller than the targeted delay, it will ensure that the user can utilize the resources well for delivering the packet within the time period. In the scheduling process, the user can reach the enhanced fairness index and throughput. Whenever the user count increases, the resources can also increase in parallel. The transmission rate demonstrates that the source can transmit the group of packets without receiving the acknowledgements. The active connection contains the roundtrip time for transmitting the data packets. The maximum throughput has been achieved for allocating the resource block. A resource block is the tiny unit of resources which could be utilized to the user equipment. The resource block has the parameters of time, frequency with subcarriers for capturing the signals. LTE frame contains the time unit-based parameters like frame, subframe, half frame, symbol, slot, and basic time unit. The frequency units are related with the total amount of sub-carriers and resource blocks. The carrier for implementing the LTE frame is the resource component. It has the value of data with single complex value for producing the data from the signal. User data are converted into a carrier modulation format along with the time domain symbols, which are converted into the frequency domain. For implementing the frequency domain, the downlink and the uplink frames are divided by the frequency with continuous and synchronous transmission. 

## 3. Experimental Results

### 3.1. Experimental Setup

In this section, we compare the proposed ITFDS scheduling algorithm with some related methods, namely PSS [35] and QuAS [39], for real-time and non-real-time applications. Every user equipment packet could be scheduled to the queue for transmission through the proposed LTE downlink scheduling technique. The frequency domain is used for the utilization of bandwidth in an efficient manner. Table 4 shows parameters for simulation.

### 3.2. Packet Delay Verses Different UEs

**Figure 5:**
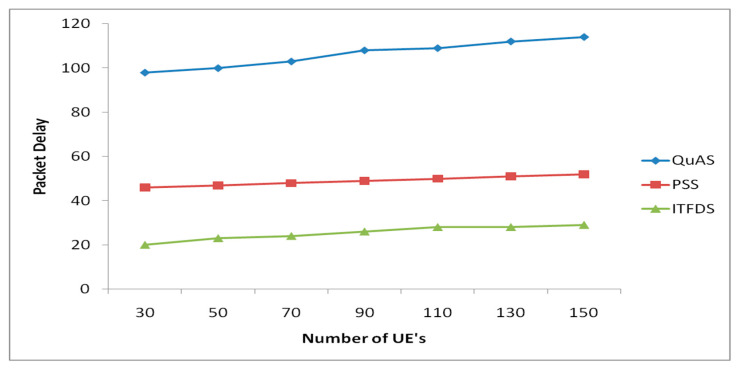
Packet delay versus different UEs.

The packet delay has been predicted using the enhanced technique through the queue in the LTE networks. The resource blocks are effectively utilized for providing the frequency-based time allocation strategy. The virtual queue has been framed to analyze the total amount of incoming packets and used to modify the packets for transmission also to satisfy the delay needs. A gradient related methodology is implemented to minimize the optimization problem for every time period. Figure 5 shows the performance of packet delay verses different UEs in the simulation. The results of the simulation indicate that, as long as w_k_ and tth are properly adjusted, the proposed scheduler can cope well with both RT and NRT traffic simultaneously. In addition, the proposed scheduler also ensures a certain level of fairness among all the users because it is based on the PF criterion. The results of the simulation also show that the delay is increasingly as much as users are getting increase and PF suffers a largest delay in the network compares to QuAS, PSS and ITFDS. The proposed technique shows better performance than QuAS and PSS, because of different weight is allocated for different applications. QuAS and PSS allow only fair allocation so that the same weight is distributed to all UEs. The proposed ITFDS algorithm distributes various delays to different applications but it always gives the best performance compared to the others because of combination both time domain and frequency domain to scheduling. In the simulation, proper setting of weight value w_k_ can reduce the delay effectively. As the result, the delay in ITFDS does not exceeds 5 ms. For high system load, the maximum of the mean RT packet delay was always equal to 0.1 s because the RT packet would be dropped if it violated the threshold of 20 rounds (i.e., 0.1 s). 

### 3.3. Packet Loss Ratio Verses Different UEs

**Figure 6:**
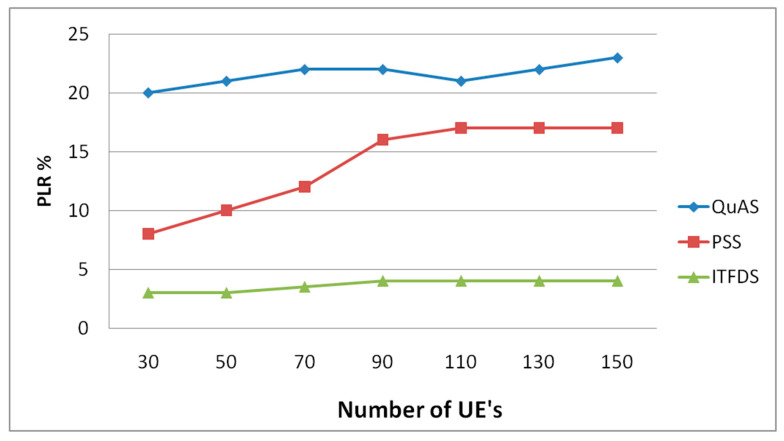
Packet loss ratio versus different UEs.

Figure 6 shows the packet loss rate for various scheduling algorithms. Packet loss rate (PLR) is one of the major parameters in wireless communication that directly affects transmitted video quality. The proposed scheduling algorithm achieves lower PLR than other scheduling algorithms. The proportional fair algorithm has the highest packet loss ratio. When the speed of the UEs is increased, the PLR of QuAS and PSS are also increased. The proposed algorithm maintains the same PLR for all the various speed of UE. The packet loss ratio may impact huge effect for transmitting large amount of data. The scheduler has not been affected while the total amount of users increased. The existing methods did not provide the priority for the packets while performing the scheduling process. On the contrary, the proposed technique has the implementation of packet priority for transmission so that the packet loss ratio is the minimum value for the proposed technique compared to the relevant ones.

### 3.4. Aggregated Throughput Verses Different UEs

**Figure 7:**
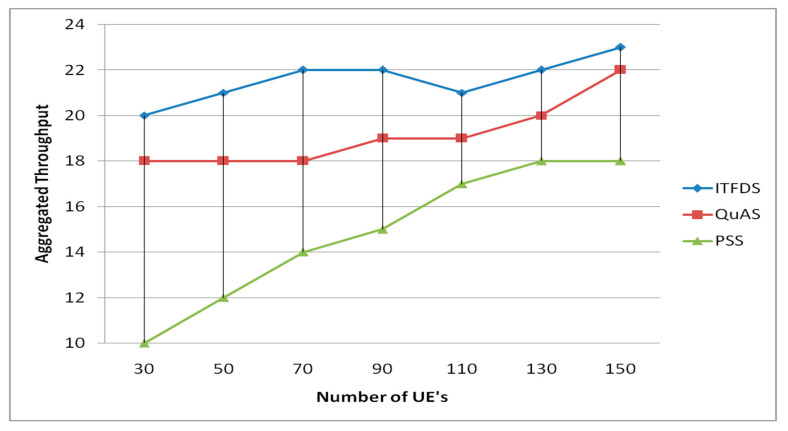
Aggregated throughput versus different UEs.

Figure 7 shows the aggregated throughput for various applications. As the result, the proposed method does not show better performance inside of aggregated throughput compared with the others because in non-real time applications, the change in the weight does not have any impact. The choice of data flow is improved according to channel quality indicator (CQI) with low traffic demand. Since video transmission depends on both QCI and CQI, changes in α value lead to variation in the video transmission. Figure 7 shows the throughput performance for various applications by varying the value of α. Improvements of the proposed method will be further investigated in future works.

### 3.5. Fairness Index Verses Different UEs

**Figure 8:**
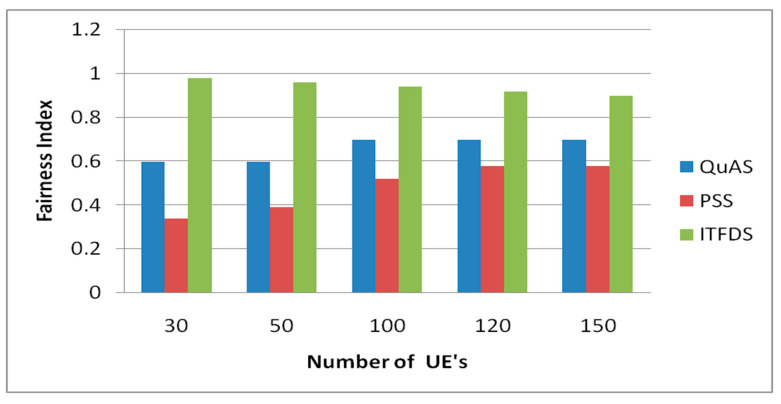
Fairness Index versus different UEs.

Figure 8 shows the fairness index for various UEs. From the obtained results, the QuAS and PSS mostly perform in a similar way. The main distinction between these two methodologies is convolution. Using the proposed ITFDS scheduling, the complexity is reduced, and the fairness is improved. The fairness index is computed using the values of normalized throughput and the total number of dynamic connections in LTE. The fairness is another parameter for producing the quality of service, the user service requests are very essential to transit the large amount of data. The fairness value has been increased in the proposed technique that allocates more amounts of resources whenever the total users are increased.

### 3.6. Comparison of Spectral Efficiency and Overhead

**Figure 9:**
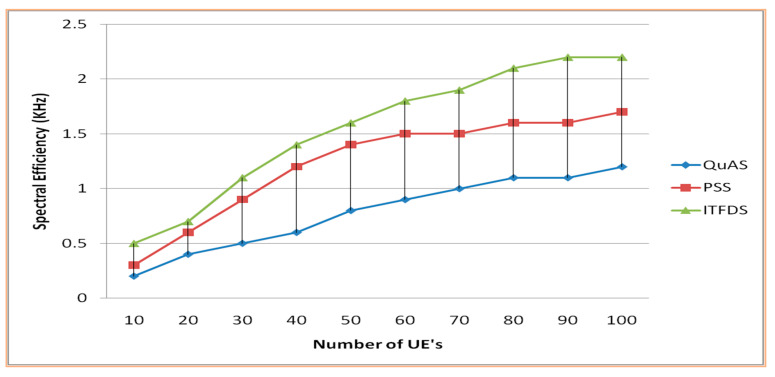
Spectral Efficiency versus different UEs.

The proposed scheduling technique is compared with the related methods, namely QuAS and PSS, according to the performance metrics of spectral efficiency and overhead. The proposed scheduling algorithm has been implemented in eNodeB to enhance the spectral efficiency with guaranteed quality of service. Figure 9 demonstrates that the proposed technique has better performance regarding the amount of spectral efficiency compared to the PSS and QuAS techniques. The result of the simulation shows that, when the number of users is small (10 as in simulation), there were a small difference between these algorithms, but when the number of users is increasing, the proposed method shows better results than the other. The quality of the transmission through the channel for the user depends on the transmission range and the obstacles within the base station and the user. The spectral efficiency is computed to estimate the throughput of every user according to the SINR value in eNodeB and the quality of the signal.

**Figure 10:**
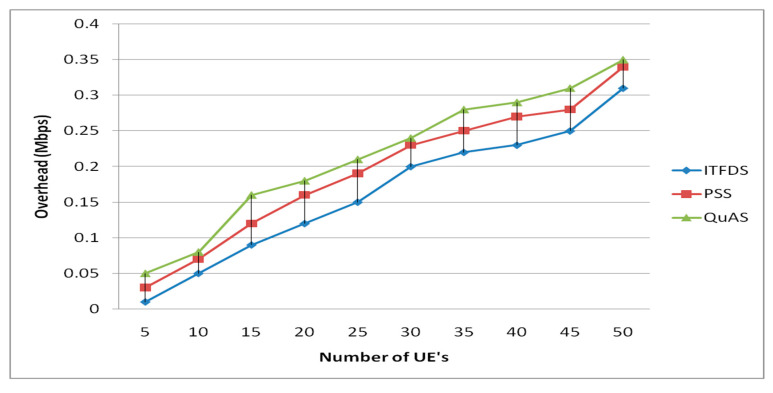
Overhead versus different UEs.

In the simulation, the overhead while eNodeB transmits packets using the proposed LTE downlink scheduling technique is also observed. It is directly linked with the channel bandwidth for providing efficient transmission in LTE. The small amount of overhead demonstrates a better quality of service. Figure 10 illustrates the proposed scheduling technique has minimized overhead compared to the related techniques.

### 3.7. Complexity Analysis

The complexity analysis of the proposed method is computed as follows. Let *n* be the total amount of users, every resource block is used to transmit the packet through LTE. Every time the user can select the improved rate to utilize the resources for transmitting data packet. If the proposed technique identifies *p* users from the total amount of users, the complexity for the time period is O(logp). The mean rate is used for selecting per user as O(1). The resource block (RB) is responsible for identifying the capable user to transmit the packets. The average arrival time is computed and analyzed the final transmitted packet in the active queue. The estimated time is also computed within the specified time period. The probability through the data transmission is calculated for analyzing the time complexity in the queue. The scheduling process is also analyzed for transmitting the data packet, and the overall time complexity is computed as O(logp)  in the resource block.

## 4. Conclusions

In this paper, we have proposed a new channel-aware integrated time and frequency-based downlinks LTE scheduling (ITFDS) algorithm. The proposed scheduling algorithm distributes the resources to all the users together using time-based and frequency-based scheduling. It works well for real-time flows and also maintains fairness among users. ITFDS outperforms other scheduling strategies in terms of system throughput, fairness and packet loss ratio. The simulation environment included both GBR and non-GBR bearers. The proposed scheduling algorithm achieved the QoS requirement of GBR bearers such as multimedia applications with increased system throughput and provided fairness to non-GBR bearers. The packet loss rate of the proposed scheduling algorithm has been reduced significantly compared to other scheduling strategies. 

Although it outperforms other algorithms, the ITFDS algorithm does not have good enough performance compared against other algorithms in terms of aggregated throughput. The proposed method is also deployed for 4G-LTE, but not for 5G technologies. The proposed LTE has some downside for implementing real-time applications in that LTE data rates minimize whenever the distance from the tower to the user equipment is very long. Sometimes, connectivity problems occur when voice is being converted into the packets, so that the transmission time may increase. Thus, our aims in future works are to investigate the extensions of the scheduling algorithm to other contexts and with 5G technology to ensure it is good enough to apply in Industry 4.0.

## Figures and Tables

**Table 1:** The notions and symbols used throughout the paper

Symbol	Meaning
CQI	Channel Quality Indicator
QCI	QoS Class Identifiers
DCE	Dynamic Class-based Establishment
QoS	Quality of Service
PF	Proportional Fair
MT	Maximum Throughput
TTI	Transmission Time Interval
MWLDF	Maximum Weight Largest Delay First
EXP/PF	Exponential-Proportional Fair
$\mathrm{Cy}{\left(n\right)}_{i,k\;}$	Maximum limit of the transmission rate
$C_{i,k}$(t)	Current channel condition
$\bar{C_{i}}$	Mean data rate for *i*th user
$R_{i,k}^{\mathrm{PF}}\left(n\right)$	Throughput
HOL	Head of Line
$\Delta_{i,k}$	Delay
$W_{i\;}\left(t\right)$	Weighted time domain value
$Q_{i,k\;}$	Buffer size
PLR	Packet Loss Rate
UE	User Equipment
SINR	Signal-to-Interference-Noise-Ratio
GBR	Guaranteed Bit Rate
non-GBR	Non-Guaranteed Bit Rate
H2H	Heart-to-Heart devices
M2M	Machine-to-Machine devices
T	Queue-based transmission matrix
k	State
$\delta_{k}$	Transmission state probability of the state k.
R(Q)	Resultant length of the class in the queue
$P_{c}$	Parameter of channels
$T_{v}$	Transmission rate
$T_{w}$	Weight of the transmission range
$T_{\mathrm{beginning}}$	Beginning transmission range
$T_{\mathrm{current}}$	Current transmission range

**Table 2:** Standard QCI Characteristics defined in LTE (Release 13)**.**

QCI	Type of Resource (00-GBR, 01- non-GBR)	Priority of QCI	Priority Delay (ms)	Packet Loss Ratio	Traffic Type
1	00	2	100	10^−2^	Speech Signal
2	00	4	150	10^−3^	On-line Video Streaming
3	00	3	50	10^−4^	Buffered Video Streaming
4	00	5	50	10^−3^	Real-time Applications
5	01	0.7	300	10^−4^	IMS Signaling
6	01	2	100	10^−3^	Video Streaming
7	01	1	300	10^−4^	Buffered Video
8	01	6	300	10^−4^	Transmission Control
9	01	7	300	10^−4^	Protocol-based

**Table 3:** Standard CQI.

CQI	Modulation Type (00- QPSK, 01-16 QAM, 02-64 QAM)	Actual Coding Rate	Required SINR
1	00	0.07618324	−4.46
2	00	0.11795332	−3.75
3	00	0.18848214	−2.55
4	00	0.30078125	−1.15
5	00	0.48730469	1.75
6	00	0.58789063	3.65
7	01	0.36914063	5.20
8	01	0.47851563	6.10
9	01	0.60156250	7.55
10	02	0.45507813	10.85
11	02	0.57324219	11.55
12	02	0.65039063	12.75
13	02	0.75390625	14.55
14	02	0.85253906	18.15
15	02	0.96093751	19.5

**Table 4:** Parameters for the simulation.

Available Bandwidth	20.0 MHz
Amount of RB’s	100 (12.0 sub-carrier per PRB)
Cell assortment	2 Km
Number of UE’s	30,40,50, 70, 90,110,130,150
Model	Random
Speed	3km/hr,30Km/hr,120 km/hr
GBR Flows	8.4kbps for voice and H.264 for videos
Non GBR Flows	12 Kbps
Simulation time	180 s
Simulation round	100
Head of Line Delay (HOL)	0.1 s
UE speed limit	100 km/h
Bandwidth	25 MHz
Total amount of eNodeB	3
Duration	55 TTI

## References

[1] Sahu D.P., Singh K., Manju M., Taniar D., Tuan L.M., Son L.H., Abdel-Basset M., Long H.V. (2019). Heuristic search based localization in mobile computational grid. IEEE Access.

[2] Yang S., Wang C., Jiang C. (2018). Centron: Cooperative neighbor discovery in mobile Ad-hoc networks. Comput. Netw..

[3] Condoluci M., Mahmoodi T. (2018). Softwarization and virtualization in 5G mobile networks: Benefits, trends and chall, nges. Comput. Netw..

[4] Wang Y.C., Chuang C.A. (2015). Efficient eNodeB deployment strategy for heterogeneous cells in 4G LTE systems. Comput. Netw..

[5] Adasme P., Lisser A. (2016). Uplink scheduling for joint wireless orthogonal frequency and time division multiple access networks. J. Sched..

[6] Nadeem M., Li Z., Malik A., Biglari-Abhari M., Salcic Z. (2019). Allocation and scheduling of System J programs on chip multiprocessors with weighted TDMA scheduling. J. Syst. Archit..

[7] Liu S., Guan N., Ji D., Liu W., Liu X., Yi W. (2019). Leaking your engine speed by spectrum analysis of real-Time scheduling sequences. J. Syst. Archit..

[8] Priya L.R., Soundar K.R. (2018). LTE: An enhanced hybrid domain downlink scheduling. Cogn. Syst. Res..

[9] Nasralla M.M., Khan N., Martini M.G. (2018). Content-aware downlink scheduling for LTE wireless systems: A survey and performance comparison of key approaches. Comput. Commun..

[10] Maia A.M., Vieira D., de Castro M.F., Ghamri-Doudane Y. (2016). A fair QoS aware dynamic LTE scheduler for machine-to-machine communication. Comput. Commun..

[11] Nasralla M.M. (2020). A Hybrid Downlink Scheduling Approach for Multi-Traffic Classes in LTE Wireless Systems. IEEE Access.

[12] Sadiq B., Baek S.J., De Veciana G. (2011). Gustavo, Delay-optimal opportunistic scheduling and approximations: The log rule. IEEE ACM Trans. Netw..

[13] Cao J., Ma M., Li H., Ma R., Sun Y., Yu P., Xiong L. (2019). A Survey on Security Aspects for 3GPP 5G Networks. IEEE Commun. Surv. Tutor..

[14] Jalali A., Padovani R., Pankaj R. Data Throughput of CDMAHDR a High Efficiency-High Data Rate Personal Communication Wireless System. Proceedings of the IEEE 51st Vehicular Technology Conference Proceedings.

[15] Beh K.C., Armour S., Doufexi A. Joint Time-Frequency Domain Proportional Fair Scheduler with HARQ for 3GPP LTE Systems. Proceedings of the 2008 IEEE 68th Vehicular Technology Conference.

[16] Samia D., Ridha B. (2015). Comparative study of Down Link Packet Scheduling for LTE Networks. Wirel. Pers. Commun..

[17] Piro G., Grieco L., Boggia G., Fortuna R., Camarda P. (2011). Two-level downlink scheduling for real-time multimedia services in LTE networks. IEEE Trans. Multimed..

[18] Liu B., Tian H., Xu L. An efficient downlink packet scheduling algorithm for real time traffic in LTE Systems. Proceedings of the IEEE Consumer Communications and Networking Conference.

[19] Wang C., Huang Y.C. (2014). Delayed Scheduler coupled throughput fairness resource allocation algorithm in Long Term Evaluation Wireless Networks. IET Commun..

[20] Zaki Y., Wardane T.W., Gorg C., Timm-Ciel A. Multi QoS Aware Packet scheduling for LTE. Proceedings of the IEEE Vehicular Technology Conference.

[21] Lai W.K., Tang C.L. (2013). QoS Aware downlink Packet Scheduling for LTE Networks. Comput. Netw..

[22] Chadchan S.M. (2013). A fair Downlink Scheduling algorithm for 3GPP LTE Networks. IJ Comput. Netw. Inf. Secur..

[23] Kwan R., Leung C. (2010). A survey of scheduling and interference mitigation in LTE. J. Electr. Comput. Eng..

[24] Pramudito W., Alsusa E. (2014). Confederatrion based RRM with proportional fairness foe soft frequency reuse LTE Networks. IEEE Trans. Wirel. Commun..

[25] Holma H., Toskala A. (2009). LTE for UMTS OFDMA and SC-FDMA Based Radio Access.

[26] Schwartz S., Mehlfuher C., Rupp M. Throughput Maximizing multiuser scheduling with adjustable fairness. Proceedings of the IEEE International Conference on Communications.

[27] Cisco Global Mobile Data Traffic Forecast Update, 2010–2015.

[28] Wang C.Y., Hsieh S.Y. (2015). Service Differentiated downlink flow scheduling to support QoS in LTE. Comput. Netw..

[29] Hussain S. (2009). Dynamic Radio Resource Management in 3GPP LTE.

[30] GPP, Tech. Specif, Specif. Group Radio Access Network Requirements for Evolved UTRA (E-UTRA) and Evolved UTRAN (E-UTRAN), 3GPP TS 25.913. https://portal.3gpp.org/desktopmodules/Specifications/SpecificationDetails.aspx?specificationId=1342.

[31] Kumar P., Kumar S., Choudhary R., Mandal J., Auluck N., Nagarajaram H. (2016). Performance Analysis of Downlink Packet Scheduling Algorithms in LTE Networks. Advanced Computing and Communication Technologies.

[32] Thienthong P., Teerasuttakorn N., Nuanyai K., Chantaraskul S. (2019). Comparative Study of Scheduling Algorithms and Almost Blank Subframe for LTE HetNets. 2019 7th International Electrical Engineering Congress (iEECON).

[33] Liu S., Zhang C., Zhou Y., Zhang Y. (2015). Delay-Based Weighted Proportional Fair Algorithm for LTE Downlink Packet Scheduling. Wireless Pers. Commun..

[34] Adesh N.D., Renuka A. (2019). Adaptive downlink packet scheduling in LTE networks based on queue monitoring. Wirel. Netw..

[35] Salman M.I., Mansoor A.M., Jalab H.A., Sabri AQ M., Ahmed R. (2017). A Joint Evaluation of Energy-Efficient Downlink Scheduling and Partial CQI Feedback for LTE Video Transmission. Wirel. Pers. Commun..

[36] Uyan O.G., Gungor V.C. (2019). QoS-aware LTE-A downlink scheduling algorithm: A case study on edge users. Int. J. Commun. Syst..

[37] Feng F., Peng F., Yan B., Lin S., Zhang J. QoS-Based LTE Downlink Scheduling Algorithm for Smart Grid Communication. Proceedings of the 2017 9th IEEE International Conference on Communication Software and Networks.

[38] Kayali M.O., Shmeiss Z., Safa H., El-Hajj W. Downlink Scheduling in LTE: Challenges, Improvement, and Analysis. Proceedings of the 2017 13th International Wireless Communications and Mobile Computing Conference (IWCMC).

[39] 3GPP Specification. https://www.3gpp.org/DynaReport/SpecReleaseMatrix.htm.

[40] Land W.H., Schaffer J.D. (2020). The Support Vector Machine. The Art and Science of Machine Intelligence.

